# Mitigating Home Environmental Asthma Triggers in Subsidized Housing: Experiences of Caregivers and Healthcare Workers

**DOI:** 10.3390/healthcare14020150

**Published:** 2026-01-07

**Authors:** Meirong Liu, Jae Eun Chung, Janet Currie, Irene Park, Dharmil Bhavsar, Sarah Ali Carlis, Imani Cabassa-George, Kyaus Washington, Minxuan Lan

**Affiliations:** 1 School of Social Work, Howard University, 601 Howard PL, Washington, DC 20059, USA; 2 School of Communications, Howard University, 1250 U Street, Washington, DC 20009, USA; jaeeun.chung@howard.edu (J.E.C.); imani.cabassa@bison.howard.edu (I.C.-G.); 3 Center for Health Wellbeing, 185A JRR Building, Princeton University, Princeton, NJ 08540, USA; 4 119 Lewis Thomas Laboratory, Department of Molecular Biology, Princeton University, Washington Road, Princeton, NJ 08544, USAdb0299@princeton.edu (D.B.); 5 Department of Geography and Planning, The University of Toledo, Toledo, OH 43606, USA

**Keywords:** pediatric asthma, subsidized housing, environmental health disparities, healthy housing, qualitative research

## Abstract

**Background/Objectives:** Pediatric asthma remains a pressing public health issue, especially among low-income, minority children living in subsidized housing. **Methods:** This study employed a community-based participatory research approach to explore barriers and potential solutions for improving asthma management in this vulnerable population. Semi-structured interviews were conducted with 22 caregivers of children with asthma and 8 community health workers in Washington, DC—a city marked by high childhood asthma rates and concentrated subsidized housing. **Results:** Thematic analysis identified six core findings: (1) families frequently encountered multiple home environmental asthma triggers, including pests, mold, secondhand smoke, leaks, poor ventilation, and aging infrastructure; (2) healthy housing services were under implemented, often due to unresponsive landlords, inadequate inspections, and poor maintenance; (3) existing services such as pest control, mold remediation, and smoke-free policies were ineffectively implemented; (4) challenges to service delivery included difficulties faced by landlords and structural barriers tied to geography, race, and socioeconomic status; (5) substandard housing conditions contributed to residents’ feelings of powerlessness, frustration, and distrust, with some taking legal action to address persistent hazards; and (6) participants recommended stronger housing code enforcement, sustained funding for home-based environmental interventions, housing-health liaisons, strengthened landlord accountability, support for landlords to facilitate repairs, centering families’ voices, and advocacy. **Conclusions:** This study underscores the persistent challenges caregivers face in managing asthma triggers in subsidized housing. The findings highlight the critical need for improved housing conditions, greater landlord and housing authority accountability, and policy reforms to ensure consistent, equitable, and sustainable healthy housing services that reduce pediatric asthma disparities.

## 1. Introduction

Pediatric asthma disparities remain a significant and persistent public health challenge in the United States. Asthma, the most prevalent chronic disease among American children, results in frequent emergency room (ER) visits, hospitalizations, and school absences, significantly impacting children’s quality of life and placing a substantial burden on healthcare systems [1,2]. Although asthma-related ER visits have declined nationally 2006 to 2018, higher rates persist among communities of color, low-income populations, urban residents, and those in the southeastern United States [3]. Moreover, non-Hispanic Black and Hispanic children living in subsidized housing experience disproportionately high asthma prevalence compared to their White or Asian peers [4,5]. Non-Hispanic Black children, in particular, are nearly five times more likely to be hospitalized for asthma than non-Hispanic White children [6].

Children living in subsidized housing face particularly high asthma risk. They experience higher odds of current asthma compared to children in market-rate housing or homeowners—especially among younger children and racial and ethnic minorities [5,7]. Even when considering socioeconomic and demographic characteristics, families residing in public housing are more likely to have a school-aged child with asthma compared to other low-income renters without housing assistance [8].

Asthma is uniquely sensitive to indoor environmental conditions—such as mold, pests, secondhand smoke, structural leaks, poor ventilation, dust accumulation, and aging infrastructure—that are disproportionately present in subsidized housing developments [9]. Disease-specific interventions—such as targeted home repairs, improved ventilation, and “green” housing—have demonstrated significant reductions in asthma symptoms, hospital visits, and school absences, sometimes rivaling the effectiveness of medical treatments [10]. In addition, housing code enforcement, environmental health inspections, and the implementation of smoke-free or integrated pest management policies shape how families are able to mitigate environmental asthma triggers within the home [11,12].

Building on this, we examined how housing practices and policies—particularly those intended to promote safer and healthier indoor environments—are implemented by landlords and property managers and experienced by families. Using a community-engaged, participatory approach, we conducted semi-structured interviews with caregivers of children with asthma and healthcare workers (HCWs) to understand how housing conditions contribute to asthma management challenges. This study further seeks to generate caregiver- and provider-informed recommendations to strengthen programs and policies aimed at reducing environmental asthma triggers in subsidized housing.

We conducted this research in Washington, DC, where the pediatric asthma rate (9.7%) exceeds the national average (7.0%) and public housing is highly concentrated [12,13]. Further, asthma is the most common chronic health condition among DC public school students [14].

**Figure 1:**
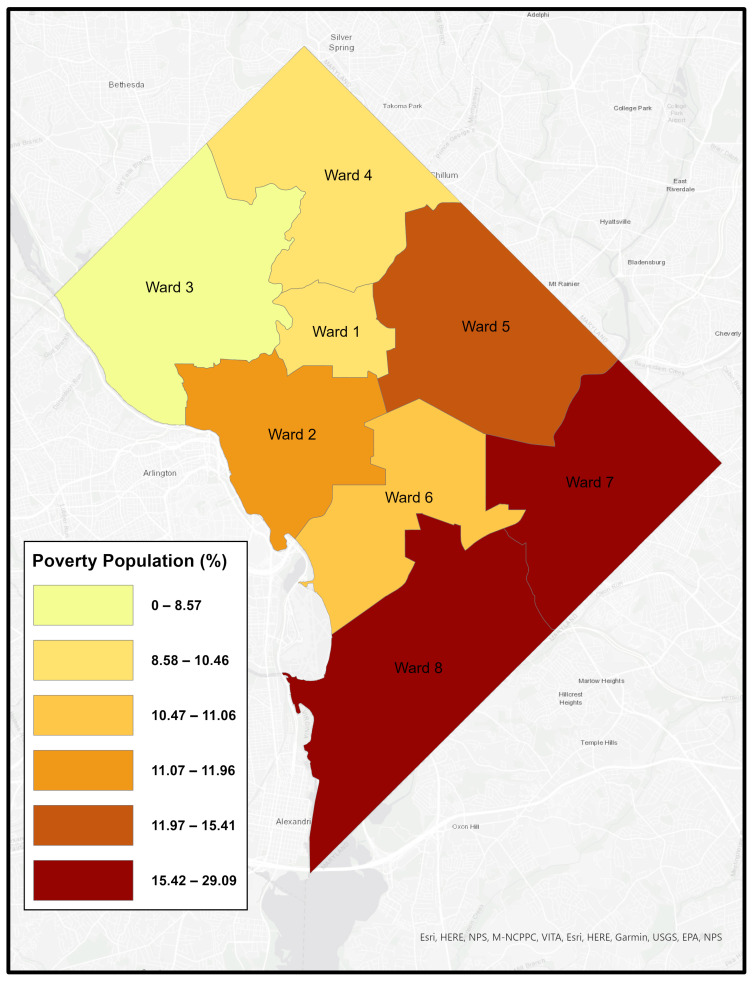
Percentage of Poverty by Ward, Washington, DC [15].

**Figure 2:**
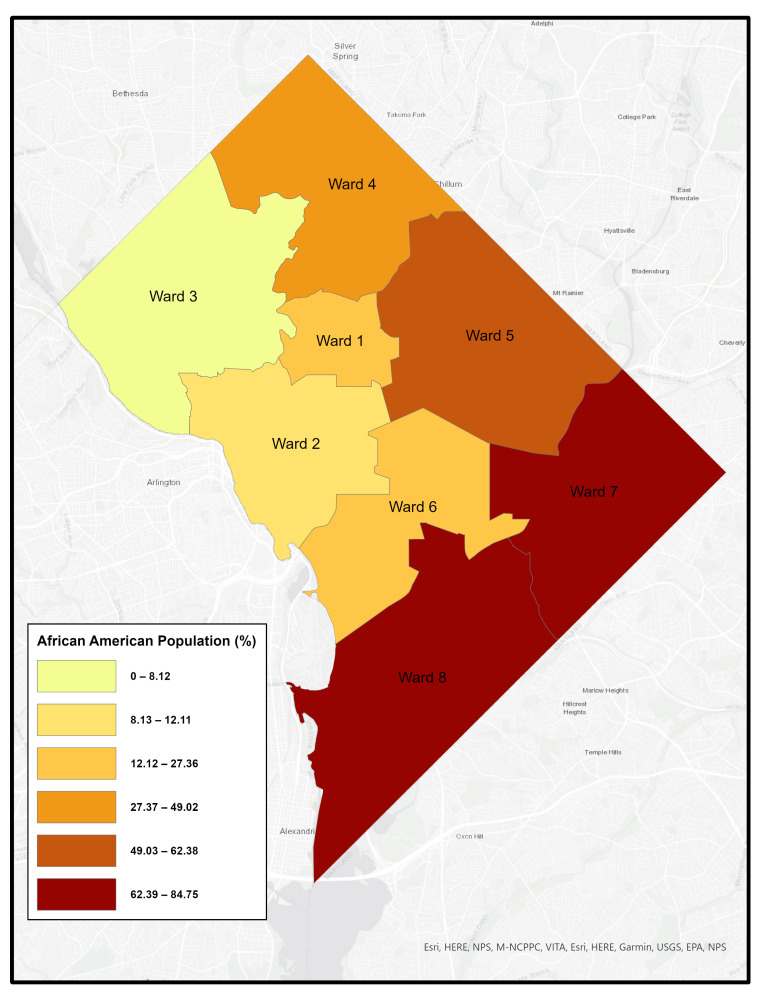
Percentage of African American Population by Ward, Washington, DC [16].

**Figure 3:**
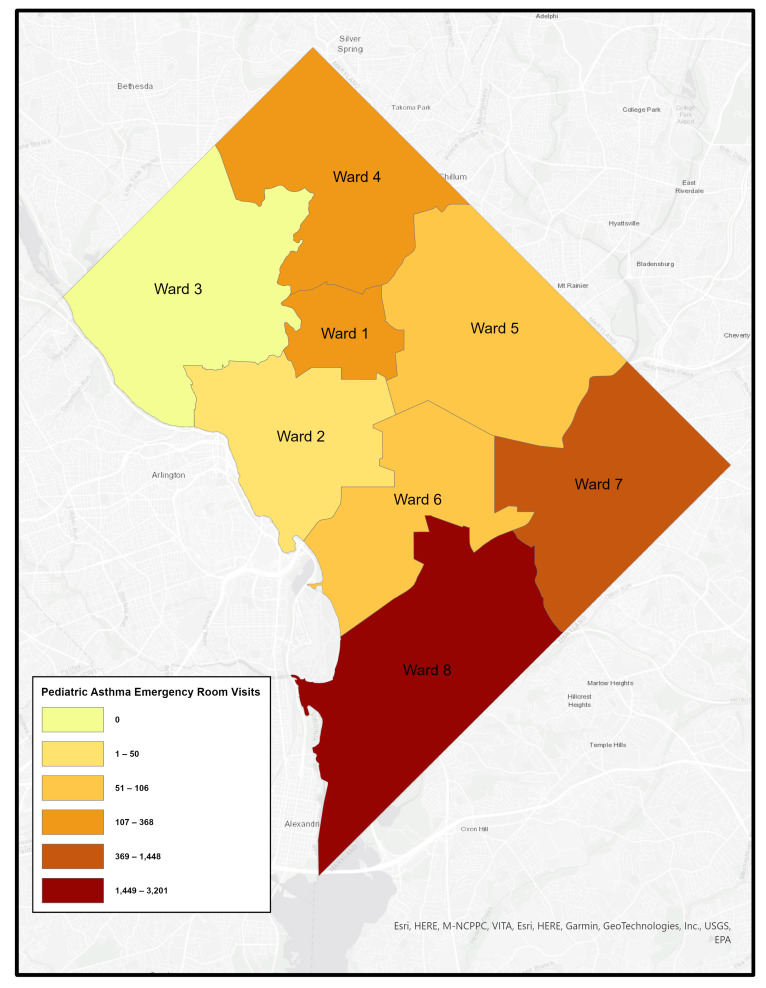
Pediatric Asthma Emergency Room Visit by Ward, Washington, DC [17].

**Figure 4:**
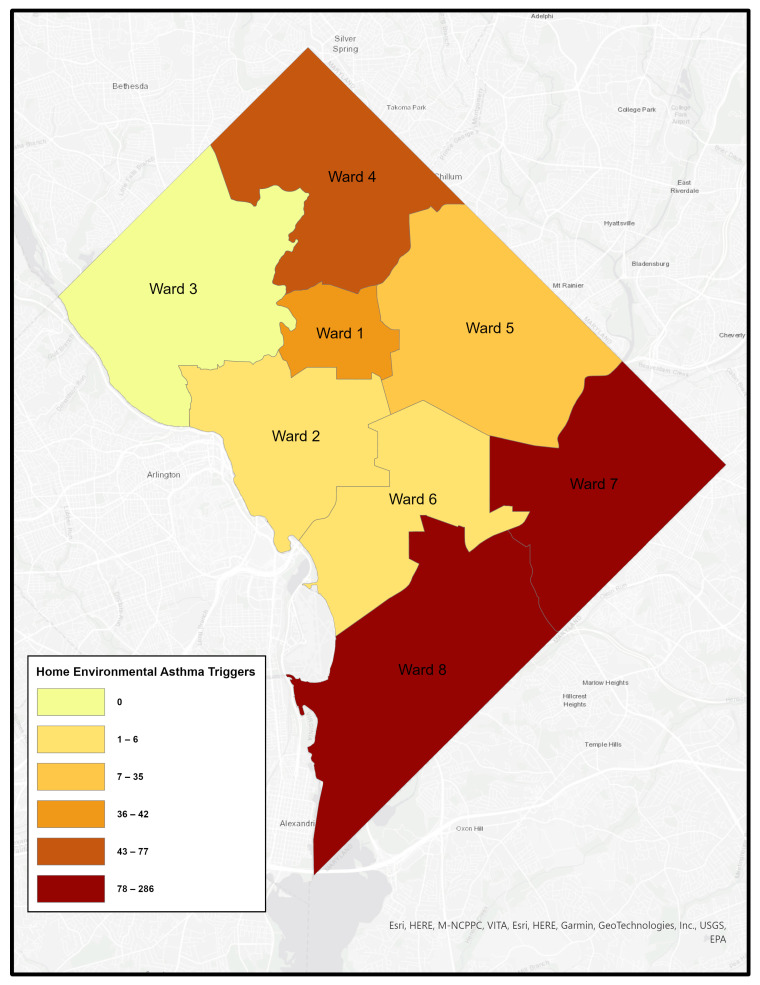
Home Environmental Asthma Triggers by Ward, Washington, DC [17].

Like many urban areas in the U.S., Washington, DC is racially and economically segregated. The city is divided into eight governmental “Wards”, with the Anacostia River separating Wards 1–6 on the west side from Wards 7 and 8 on the east. Wards 7 and 8 are predominantly African American, experience the highest poverty rates, and have long been underserved–facing environmental conditions that contribute to poor health outcomes. Figure 1, Figure 2, Figure 3 and Figure 4 illustrates ward-level patterns of poverty, racial composition, pediatric emergency department visits, and home environmental asthma triggers, respectively. Given these intersecting social, environmental, and health disparities, DC provides a critical setting to examine the challenges and opportunities for improving asthma-related housing interventions in subsidized housing.

## 2. Literature Review

### 2.1. Environmental Factors Related to Pediatric Asthma

Asthma has a complex etiology involving both genetic and environmental factors [18]. Disproportionate exposure to air pollutants in low-income communities is a well-documented manifestation of environmental injustice [19]. For example, Black Americans are consistently exposed to higher levels of particulate matter (PM2.5) than White Americans [20]. This exposure is linked to adverse birth outcomes, including low birthweight—a known asthma risk factor [21]—as well as early-life direct respiratory effects.

### 2.2. Home Environment and Pediatric Asthma

Housing conditions are key contributors to asthma disparities [9]. Home environmental triggers, such as pests, rodents, mold, mildew, cockroach droppings, poor ventilation, and secondhand smoke, are more prevalent in lower-income housing, including public and subsidized housing [22,23,24]. Indoor air pollutants—including nitrogen dioxide, particular matter, dampness, and elevated indoor temperatures—are also prevalent in subsidized housing and are associated with poor asthma control and increased ER visits [11,25]. Children in substandard urban housing face increased exposure to indoor triggers and maintenance issues, leading to higher asthma morbidity and exacerbations [26].

### 2.3. Policies and Programs Addressing Home Environmental Issues

Several policies aim to mitigate indoor hazards in subsidized housing, including smoke-free rules, green housing initiatives, and Healthy Homes (HH) programs. In 1999, the U.S. Department of Housing and Urban Development (HUD) launched the Healthy Homes (HH) Initiative [27] to address health risks in residential settings. Funding supported research and demonstration projects, and further, interventions were identified to address multiple hazards, including moisture and dust control, indoor air quality improvements, and resident education.

A joint call to action by HUD and the Surgeon General in 2009 emphasized the need to address preventable home-related diseases such as asthma and lead poisoning [28]. The HH Initiative (now Program) has since been implemented through competitive grants, demonstration projects, and interagency agreements. In FY26, the proposed federal allocation for non-lead Healthy Homes efforts totals approximately $145 million [29]. Grantees are tasked with developing low-cost hazard assessments, evaluating intervention effectiveness, building local capacity, and offering public education [30]. Many HH programs have expanded to include in-home asthma education and intervention components, though these vary in intensity, duration, types of interventions and providers, delivery model, outcome tracking [31].

### 2.4. Housing Codes

Many U.S. municipalities have adopted housing codes requiring landlords to maintain safe conditions, but enforcement is typically “report-based”—relying on tenants to initiate complaints by contacting regulatory agencies [11]. Some jurisdictions have implemented proactive code enforcement programs that require routine inspections of rental properties regardless of tenant complaints. While proactive models hold promise for improving housing conditions more equitably, report-based systems continue to predominate in many settings and often fail marginalized residents, who may hesitate to report poor conditions either for fear of retaliation from landlords or lack of awareness about reporting procedures [32].

In DC, housing codes specify minimum standards for ventilation, heating, pest control, structural soundness, and plumbing. Rental units must be pest-free, have intact floors, lead-safe paint, and leak-free plumbing. Tenants must file complaints through the Department of Buildings if landlords fail to comply. The Housing Conditions Court offers a streamlined process for enforcing violations [33].

### 2.5. Gaps in the Literature and Study Purpose

HH programs and housing code enforcement aim to reduce environmental asthma triggers, but children living in subsidized housing still face disproportionately high asthma diagnoses, ER visits, and hospitalizations compared to children in similarly low-income, but non-subsidized households [5,34]. These disparities persist alongside caregivers’ frequent reports of at-home asthma triggers [27]. For example, a survey of 226 caregivers of children with moderate to severe asthma—mostly in subsidized housing—found indoor environments inadequate for asthma management and landlords’ efforts insufficient [35]. Recent studies continue to document the persistent challenges caregivers face in managing asthma triggers in subsidized housing, including ongoing exposure to mold, pests, and inadequate ventilation despite existing programs intended to improve housing quality [9,27]. These findings suggest a disconnect between program design and real-world implementation. Substandard housing, structural defects, and inadequate landlord response make it difficult for caregivers to control these triggers, even when they are aware of the risks [9,36]. Even with educational interventions, caregivers may be unable to reduce exposure to triggers due to systemic issues such as a lack of power, resources, or support to take effective action [37]. Studies have also underscored the need for stronger accountability among landlords and housing authorities and policy reforms to ensure the consistent and equitable implementation of healthy housing services in low-income communities [11,22,38]. Collectively, sustained funding for HH programs and robust housing code enforcement, together with broader policy changes—are therefore necessary to reducing environmental asthma triggers for in low-income and minoritized communities where substandard housing is prevalent [9].

While most studies have relied on surveys to report the prevalence of home triggers, asthma-related outcomes, and intervention evaluations, few have explored the lived experience of caregivers—particularly those not receiving services from HH programs despite evident need. Understanding how these programs are perceived and experienced by families, and how landlords and property managers implement them in practice, is critical for improving interventions. Moreover, although HCWs often serve vital roles in connecting families to care, delivering asthma education, and addressing social determinants of health—including housing, their perspectives remain underexplored [39].

### 2.6. Methodological Challenges

Qualitative research with marginalized populations in subsidized housing presents unique challenges. Residents—particularly low-income women of color—often face surveillance and control by housing authorities, contributing to distrust and reluctance to participation in research [40]. Historical abuses, fear of retaliation, stigma, and safety concerns further hinder recruitment [41,42]. Geographical segregation and social isolation limit access and sustained engagement [43]. These overlapping disadvantages complicate research design and data collection, underscoring the importance of sensitivity, trust-building, and flexible methods.

Building on these challenges, the present study uses a community-engaged qualitative approach to explore how families in subsidized housing experience asthma-related housing interventions in DC—a city with high pediatric asthma rates and housing disparities. We investigate (a) caregivers’ experiences and responses to home environmental asthma triggers; (b) the implementation and effectiveness of HH services; and (c) caregiver- and HCW-informed recommendations to improve policies and programs addressing at-home triggers in subsidized housing.

## 3. Methods

### 3.1. Community-Based Participatory Research Approach

The study applied the principles of community-based participatory research (CBPR) to ensure that research questions were informed by community knowledge and aimed at actionable changes [44]. The interdisciplinary team included researchers from a historically Black college (HBC) and a predominantly white institution (PWI), with expertise spanning economics, social work, health communication, and global health. We partnered with (a) a lung health-focused community health organization and (b) a national organization supporting resident service employees and public housing residents. These partnerships provided critical support for accessing the subsidized housing community and understanding its context. We emphasized equitable collaboration—shared power and decision-making throughout the research [45,46]. Community partners served as co-researchers and contributed to study design, implementation, and interpretation.

Trust was built gradually through sustained engagement and active listening. Our team created welcoming environments where participants felt safe and empowered to share their experiences. We demonstrated solidarity by participating in community services and events. We aimed to “meet community members where they are” by creating safe spaces for dialogs and responding to community-identified needs [47].

### 3.2. Participants

Participants included caregivers and HCWs with firsthand knowledge of asthma triggers in subsidized housing. Caregivers were eligible if they lived with a child under 22 with asthma in subsidized housing in DC. HCWs were eligible if they worked with such families.

### 3.3. Semi-Structured Interviews

Semi-structured interviews were chosen as the primary data collection method for their capacity to allow researchers to explore participants’ unique experiences while maintaining the flexibility to follow up on emerging themes during the interview [48]. Interview guides were co-developed with community partners to reflect concerns raised in the literature and within the community. Interview questions focused on: (a) implementation and effectiveness of programs aimed at mitigating home asthma triggers; (b) challenges for implementing HH services; and (c) recommendations for sustainable and equitable asthma care. The guides were adapted to suit the experiences and roles of caregivers and HCWs. Participants were encouraged to share insights and suggestions beyond the pre-defined questions.

### 3.4. Data Collection

Caregivers were recruited through convenience and snowball sampling at community health events, churches, public schools, and via referrals from resident leaders and local programs. HCWs were recruited from community health centers and a pediatric asthma clinic offering asthma education and support services.

Data were collected between May 2023 to January 2024 and included 22 caregivers and 8 HCWs. The sample size was deemed sufficient upon reaching thematic saturation [49]. Interviews were conducted in-person, by phone, or via Zoom based on participant preference. Written informed consent was obtained, and all interviews were recorded. Caregivers received a $50 gift card. Interviews averaged 20 min for caregivers (range: 9–52 min) and 27 min for HCWs (range: 24–31 min). The study was approved by the university’s Institutional Review Board.

### 3.5. Data Analysis

Recordings were transcribed verbatim using Trint software [50] and checked for accuracy. Identifying information was removed to protect participants’ confidentiality. We conducted thematic analysis [51]. This method combines both inductive and deductive strategies to identify themes. Our team first familiarized ourselves with the data by conducting the interviews and reading the transcripts. During the first phase of coding, the four team members open coded interviews using Word and then compared and revised codes during researcher meetings to achieve consensus and to develop an initial code book. In the next phase, subsequent coding was conducted in NVivo 14.0 software. Each code was double-checked by at least two coders. In this coding process, a few new codes emerged and were added. Finally, we used a finalized code book to craft the analytic narrative and select representative interview excerpts.

Rigor and trustworthiness were ensured through peer debriefing, memoing, and maintaining a detailed audit trail [52]. Experts reviewed the interview guides prior to the first interview to ensure dependability. We maintained a detailed audit trail documenting the entire data collection process. We kept field memos and engaged in peer debriefing among researchers to strengthen the credibility of the findings. Community partners also reviewed and verified the accuracy and interpretation of findings. Further, preliminary results were presented to resident service staff in affordable and assisted housing at a national conference. Feedback from the resident staff—who directly involved in subsidized housing management and advocacy—ensured our findings resonated with the residents’ lived experiences. Community partners brought perspectives from real-world scenarios, leading to a more nuanced and accurate interpretation. Collaborating with them revealed insights that researchers alone might miss, resulting in a richer, more credible analysis [53]. Finally, we provided “thick description” to maximize participants’ input, resulting contextually grounded insights that support the transferability of findings across different settings [54]. Verbatim quotes illustrate key themes and add depth to our findings.

### 3.6. Participants’ Characteristics

**Figure 5:**
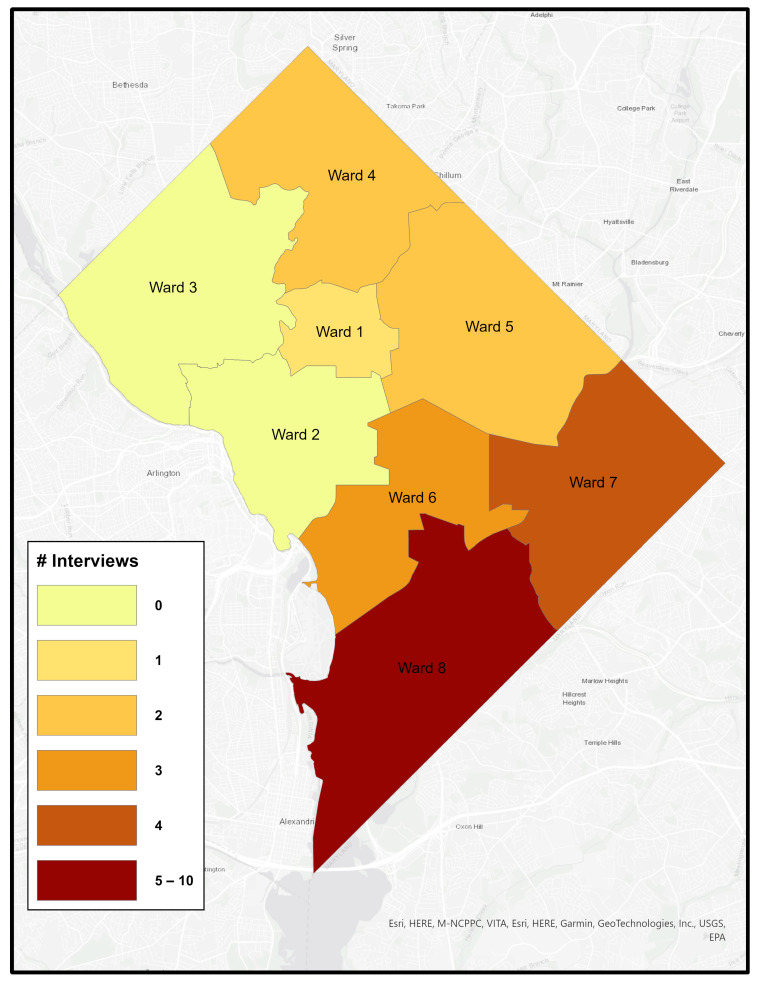
Distribution of Caregiver Interview Participants by Ward, Washington, DC.

All caregivers had children with asthma in subsidized housing. All but one were female and African American, with one identifying as Hispanic. All but two were mothers; one father and one grandmother participated. Fourteen (63.6%) resided in Housing Choice Voucher Program (HCVP) units, six (27.3%) in public housing, and one (4.5%) in a shelter (previously in HCVP). The average number of children in each household was 2.38 (*SD* = 1.40; Median = 2; Range = 1 to 5). Each household reported having one asthmatic child (*M* = 1.19; *SD* = 0.40; Median = 1; Range = 1 to 2). Figure 5 shows that the majority of caregiver participants lived in underserved areas east of the Anacostia River: nearly half (*n* = 10) lived in Ward 8, followed by Ward 7 (*n* = 4), Ward 6 (*n* = 3), Wards 4 and 5 (*n* = 2 each), and Ward 1 (*n* = 1).

All HCWs were female; six identified as African American (75.0%) and two as Hispanic (25.0%). Five were clinical asthma educators (62.5%) and three were community health workers (37.5%). Three (37.5%) reported having worked on healthcare for 1 to 2 years, one (12.5%) for 4 to 5 years, and three (37.5%) for over 5 years.

## 4. Results

Six themes were identified: (a) families commonly faced multiple asthma triggers at home, including pests, mold, second-hand smoke, leaks, poor ventilation, and deteriorating infrastructure; (b) healthy housing services were under-implemented often due to unresponsive landlords, inadequate inspections, and poor maintenance; (c) existing interventions—such as mold remediation, pest control, and smoke-free policies—were often ineffectively implemented; (d) service delivery was hindered by challenges faced by landlords and structural barriers tied to geography, race, and socioeconomic status; and (e) substandard housing conditions contributed residents’ feelings of powerlessness, frustration, and distrust, with some pursuing legal action to address persistent hazards; and (f) participants recommended stronger code enforcement, sustainable funding, strengthened landlord accountability, housing-health liaisons, reinforce landlord accountability, support for landlords to facilitate repairs, centering families’ voices, and advocacy. A summary of these themes with illustrative participant quotes is presented in Table 1.

### 4.1. Theme 1: Prevalence of Home Environmental Asthma Triggers

Both caregivers and HCWs identified the prevalence of multiple home environmental asthma trigger issues, such as pest infestations, mold, water leaks, poor ventilation, secondhand smoke exposure, carpeting, and aging infrastructure, as well as their impact on pediatric asthma symptoms. HCWs further highlighted that the existence of these triggers could undermine the effectiveness of clinical asthma management.

#### 4.1.1. Caregivers’ Perspectives

*Prevalence of Home Environmental Asthma Triggers:*

Most caregivers described rodents, roaches, and other insects as home environmental hazards. One caregiver shared, “I caught one (rat) and another jumped over” (CG 103), while another described, “The house was infested with mice, cockroaches, everything you could think of flying” (CG 104).

Persistent mold—particularly in bathrooms and basements—was another common concern: “The bathroom? The mold was everywhere” (CG 108); and “Yes (we observe dark mold)” (CG 106)

Issues with carpeting, water leaks, poor ventilation, and peeling paintings were reported. “The carpet triggered the boy’s allergies” (CG120). “I’ve got a leak in my property” (CG 102), and another describing the removal of drywall due to ongoing kitchen leaks (CG 108). Most of the caregivers indicated a functional heating system but poor ventilation remained a concern.

Several caregivers linked asthma triggers to aging infrastructure. “The building has been up since the thirties” (CG 107). “It used to be a drug and alcohol program… they remodeled it years ago” (CG109). Structural problems such as plumbing issues and poor air quality were noted: “it’s a plumbing issue inside my building unless they tear the crap down” (CG 112); and “we can’t barely breathe in it” (CG106).

*Secondhand Smoke Exposure:*

Many described exposure to secondhand smoke. One caregiver explained that the biggest issue for her was “having to walk the kids through the building full of smoke and stuff” (CG 120). Another explained: “the houses are close together. So, if someone else is [smoking] at [at home], it comes outside; the child gets the whiff of the smoke” (CG 101). Others emphasized “We have a tobacco building in the back”. A caregiver noted the second-hand smoke exposure in their building: “Dealing with the environment—where we live, people like to smoke a lot. So, it triggers it (asthma) a lot. It can be very stressful for the three of us to walk through that stuff to get to our apartment” (CG 108).

*Impact on Children’s Asthma Health:*

Caregivers linked housing-related environmental hazards to serious health comes in their children. A mother shared “He [her son] was in the ICU for asthma. I think that the housing conditions have something to do with it” (CG 108).

#### 4.1.2. Healthcare Workers’ Perspectives

*The Prevalence of Home Environmental Asthma Triggers:*

One HCW explained “Parents will often talk about pests… cockroaches, mice, water damage and mold are common triggers. Carpet removal is also a major one. Old and just dingy carpet carries a lot of dust and dust mites” (HCW 7). One described the pest infestations encountered during virtual home visits “I did a virtual home visit before the grant ended. And parents open certain cabinets or closets, and then you’ll see some roaches like out” (HCW 6).

*Impact on Children’s Asthma Health:*

HCWs echoed the impact of home environmental asthma triggers. As one provider explained: “Definitely the home environment plays a huge role in asthma outcomes. The presence of carpet, high amount of dust, pests like cockroaches or mice, and water damage are more likely to lead to asthma exacerbation for children” (HCW 4).

*Clinical Limitations Amid Home Environmental Hazards:*

HCWs further emphasized that substandard housing environments often undermine the effectiveness of clinical cares. As one explained: “It’s really hard to treat families and then send them home when they have a lot of exposure. It gets very challenging knowing that there’s only so much you can do to help out a family” (HCW 2).

### 4.2. Theme 2: Gaps in Implementation of Healthy Homes Services

Most caregivers were unaware of HH programs and identified unresponsive landlords, inadequate inspections and poor maintenance as key challenges. HCWs echoed the difficulties families face in addressing them.

#### 4.2.1. Caregivers’ Perspectives

*Awareness of HH Services:*

While some had heard of specific policies and programs such as smoke-free policies and pest control, most of the caregivers had not heard about “HH” programs, and comprehensive knowledge of available services was lacking. For example, many simply stated, “No, I’ve never heard of that” (CG 108).

*Landlords’ Lack of Responses and Lack of Follow-ups to Resident Requests:*

Caregivers frequently described that their requests to address home environmental hazards were often ignored, with little to no follow up to ensure issues were addressed. As one caregiver shared, “I’ve sent him lists and he never came to do anything” (CG 117). Another commented: “They [landlords] are not consistent with the follow-through… They don’t follow up to ensure that the things get done” (CG 112).

*Inadequate Inspections, Pest Control, and Air Filter Replacements:*

Caregivers reported missing or gaps regarding required home inspections, pest control, and air filter replacement. One commented: “Housing does a once-a-year inspection, which I haven’t had an inspection since 2019. We are about to go into 2024” (CG 118), while another said, “Sometimes they don’t even come out to inspect the unit. Sometimes they do a phone inspection” (CG 113). Pest control was described as sporadic “They have pest control on the list. But no, never have I heard about anything like that” (CG 112); and “The pest control came out about twice, but they stopped coming in” (CG 108). Air filters were often neglected: “They haven’t changed it since I’ve lived here. Probably four years… The one [air filter] upstairs definitely needs to be changed. there’s a lot of dust and I tried to wipe it.” (CG 104).

#### 4.2.2. Healthcare Workers’ Perspectives

*Landlords’ Lack of Responses:*

HCWs confirmed the unresponsiveness of landlords and property managers to caregivers’ requests: “they [families] have unresponsive landlords” (HCW 9), while others emphasized, “They have mold, but no one will come out to check it. They’ll have the same issues ongoing for a longer period… Maybe they [landlords/property managers] just don’t really care because they may not understand the conditions, they’re not living in it or they’re not experiencing the health conditions” (HCW 2).

HCWs describe this as “difficult” (HCW 9). One commented that “Families that are on vouchers tend to have the most issues have to go back and forth with the landlord and then not trying to fix things” (HCW 4). Another added, “It is really hard to get in contact, [if] I am a resident and I’ve been calling and keep calling, I’m just going to give up because it’s so many barriers” (HCW 5).

### 4.3. Theme 3: Ineffective Implementation of Healthy Homes Services

Caregivers consistently confirmed the ineffective implementation of HH services, such as pest control and mold removal services, and the lack of enforcement such as smoke-free policies. HCWs echoed these concerns and further highlighted the gaps in access and sustainability of funded HH programs.

#### 4.3.1. Caregivers’ Perspectives

*Ineffective Implementation of Pest Control:*

Several caregivers elaborated on the ineffectiveness of pest control, emphasizing that without building-wide pest management, infestations persisted. One caregiver explained, “Your house is treated for pests and the one next door to you doesn’t. Because it’s so close together, you’re still going to have them [pest] regardless” (CG 101). Another caregiver commented on repeated but superficial interventions “they [exterminators] come in, they put down stick pads or something but that wasn’t keeping all the roaches away” (CG 117).

*Ineffective Enforcement of “Smoke-Free” Policies:*

While smoke-free housing policies exist, caregivers described the challenges of enforcement. One indicated that the landlord could not do much about it: “Getting them [smokers] out of the building, it’s a process with court and paperwork” (CG 120). One stated, “That’s a terrible law because nobody abides by it. If you go to any specific property, you will still see smoke outside” (CG 102).

#### 4.3.2. Healthcare Workers’ Perspectives

*Ineffective Implementation:*

HCWs echoed caregivers’ concerns and highlighted how mold and pests were often superficially addressed. One noted: “Instead of removing it, they [landlords] just cover it, so it grows back.” She further added, “A lot of the time the building does have monthly pest control. But because it’s infestation in a building, so it keeps coming back and then some [landlords] are completely unresponsive” (HCW 6).

*Gaps in Access to Funded HH Programs:*

HCWs pointed out the lack of sustainability of funded HH programs. While these programs have been effective in mitigating home environmental asthma triggers through targeted interventions, many have been discontinued due to funding constraints. For example, one noted: “We used to be able to do more through virtual home program. Now, our only response is writing landlord letters or referring families to the law center… for D.C. families, we’re not offering referrals to specific programs now” (HCW 7).

The “Home Visiting” program, part of the HH program, was jointly implemented by the government housing and environmental agencies. Low-income families with children with moderate to severe asthma can be referred to receive home visit programs to mitigate at-home asthma triggers [55]. Yet the program was referral based and was not operating during the period of our study.

### 4.4. Theme 4: Challenges in Implementing Healthy Homes Services

Both groups identified challenges in implementing HH services. Caregivers identified challenges faced by landlords and property managers and perceived structural barriers in segregated neighborhoods. HCWs highlighted the geographic disparities within DC and lack of investment in marginalized communities.

#### 4.4.1. Caregivers’ Perspectives

*Challenges Faced by Landlords* *:*

Some caregivers described the challenges faced by the landlords, “They [landlords]’re old. How can the DC government help them, help me. They’re both in their eighties” (CG108). Caregivers also pointed to the bureaucratic challenges that landlords face. One caregiver noted “She’s [the landlord] been paying the inspection for years, but now each ward got a different agency… She can’t keep up with the new inspectors” (CG 118).

*Structural Barriers in Segregated Neighborhoods:*

Caregivers shared the barriers they face as low-income, minority residents in historically segregated neighborhoods. One stated plainly, “Being a Ward 8 resident. That’s the barrier” (CG 118). Another reflected, “I think they are doing their best to try to manage it, but it’s just apartment in general with black kids” (CG 122). Another added, “I am in Southeast, I am pretty sure uptown the resources are being used properly” (CG 112).

#### 4.4.2. Healthcare Workers’ Perspectives

*Underinvestment in Segregated Neighborhoods and Pediatric Asthma Disparities:*

One asthma educator specifically highlighted underinvestment in marginalized neighborhoods as a cause of pediatric asthma disparities: “For the lower- income families Ward 7, Ward 8, their housing issues make kids’ asthma worse” (HCW 6).

### 4.5. Theme 5: The Multilevel Impact of Substandard Housing

Substandard housing conditions and the perception of being disregarded left caregivers feeling powerless and vulnerable. They often expressed frustration and distrust. In some cases, legal referrals were considered helpful. HCWs echoed this sentiment and expressed empathy for the families.

#### 4.5.1. Caregivers’ Perspectives

*Feelings of Powerlessness and Vulnerability:*

As one caregiver stated, “Single parents with multiple issues are just disregarded.” (CG 107). “If the person living in a unit and they have kids and complain about something not being fixed in their house, they [landlords] should come out and actually look” (CG 113). “Everything is about money, and if you ain’t got enough of it. You’re not going to be seen or heard” (CG 112).

*Frustration and Distrust:*

Frustration and distrust were persistent themes in caregiver narratives. Many described landlords as negligent and exploitative: “They’re slumlords. They just take your money and don’t provide the services that’s required” (CG 107). Caregivers expressed perceived corruption: “You live in a property that is corrupt, constantly stealing funds” (CG 112).

*Legal Actions:*

Some caregivers described legal actions to address housing condition “I had to get a lawyer so that I could move” (CG120), one shared, while another reflected: “I should have taken them to court” (CG 112). Legal referrals to agencies like the Children’s Law Center helped some families resolve issues like mold (CG 108). Though relocation improved conditions for some, environmental hazards often persisted: “(It) is better now. But I do sometimes see cockroaches and mice every now and then” (CG 104).

#### 4.5.2. Healthcare Workers’ Perspectives

*Caregiver Powerlessness and Vulnerability:*

HCWs echoed caregivers’ feeling and highlighted how housing conditions constrain clinical asthma care. As one described: “I’ve had parents cry and say, ‘I’m doing all I can, but I can only do so much when I go back home, where there’s a lot of environmental things that are out of the family’s control.’ It’s emotional—for them and for me” (HCW 7).

### 4.6. Theme 6. Suggestions for Healthy Homes Services

Caregivers emphasized the need for stronger landlord accountability and improved oversight of housing authorities. HCWs recommended listening to families, ensuring sustainable funding, reinforcing landlord responsibilities through policies, providing support for both housing providers and residents, engaging in advocacy, and leverage innovating approaches.

#### 4.6.1. Caregivers’ Perspectives

*Strengthening Accountability and Oversight of Landlords:*

Caregivers emphasized the need for stronger government oversight to ensure landlords and property managers address health hazards in home environments. One caregiver explained, “If they [government agencies] stay on top of these landlords and do what they need to do then you wouldn’t have these problems inside the household” (CG 118).

#### 4.6.2. Healthcare Workers’ Perspectives

*Listening to Families’ Voices:*

As one noted, “A lot of families just need someone to call and get advice from or even to listen to them because they feel unheard a lot. Having a program that allows the parent to have a voice is very important” (HCW 2). Another pointed out the need to build communication channels, noting, “there’s a gap in the communication” (HCW 9). While others proposed liaison roles to bridge the gaps between families and housing authorities: “There needs to be someone placed in like that Section 8 office like a liaison that knows what they’re going through, but able to be that middleman and express the needs of the people they serve” (HCW 5).

*Ensuring Sustainable and Consistent Funding:*

HCWs frequently emphasized the need for sustainable funding, and city-level infrastructure for consistent HH services. One described an example of a government-funded program that provided virtual home visits, pest control, air filters, purifiers, and vacuums, noting such services: “are very helpful and save a lot of money and time,” and “if we can just intervene for these things, maybe we don’t have to take it further and get a lawyer involved” (HCW 4). The same HCW emphasized that responsibility should shift from community partners to government agencies, stating that “we’re moving past the point where the responsibility should be specifically on our community partners to find funding… and more on the city and actually establishing a budget” (HCW 4). Further, such programs should support both families and landlords. One suggested such programs could “provide things at a lower cost, and help with landlords too so that they’re not paying so much,” (HCW 9)

*Reinforcing Landlord Responsibilities through Policy:*

Another major theme centered on the need for policies that hold landlords accountable for maintaining healthy housing conditions (HCW 3). As one suggested, “The government on a policy level could help the landlord to ensure that they enforce the rules” (HCW 2), “It has to serve from the government side, really cracking down on the landlords that just weren’t responsive” (HCW 6), another elaborated: “They can be stricter on the landlords, enforcing the laws and the policies that are in place, even adding new policies that show that they need to fix housing conditions.” (HCW 2).

*Supporting Landlords and Property Managers:*

Several HCWs identified the need for city officials to collaborate with landlords to identify the barriers they face and to provide technical or financial assistance to support their participation in solutions: “Some landlords do want to fix problems. They’re not necessarily slumlords. Some of them don’t really have access to that knowledge on how they can make this better.” (HCW 4). For smaller, non-corporate-owned units, repair costs often “come out of their pocket,” making timely remediation difficult. Accordingly, the same HCW suggested that “city officials to collaborate with the landlords—seeing what barriers there are for the landlords, why they can’t get these issues fixed in a timely manner—and to see if there is some support that they can be given” (HCW 4).

*Providing Tangible Supports for Families:*

HCWs voiced a need for tangible support for families. As one participant stated, “help with trying to keep the inside, much cleaner, like the humidifier, vacuum cleaners” (CG 120). One explained, “Providing funds for families to have like new filters or new carpets and things like that” (HCW 2).

*Advocating for Families:*

Advocacy was viewed as an approach to navigate housing systems, hold landlords accountable and promote healthier environments. One health worker mentioned “Really advocate for these families, they say children are our future, we need to help them as much as we would like to help ourselves. Really staying on top of housing to prevent serious things happening to these children” (HCW 9).

*Leveraging Innovative Approaches:*

Participants proposed innovative approaches, including the use of technology. One proposed: “there could be smoke detectors, and we can detect that there’s smoking in this unit and then maybe make it reprimand” (HCW 5).

## 5. Discussions

This study explored the experiences of caregivers and healthcare workers (HCWs) concerning the home environment for children with asthma in subsidized housing. Participants described widespread exposure to environmental asthma triggers, echoing prior research showing these risks are disproportionately common in subsidized housing [12].

Caregivers reported repeated, often ignored requests for repairs—highlighting a pattern of chronic neglect. This aligns with previous findings on inadequate code enforcement and its contribution to persistent substandard housing [9,11].

These housing conditions are rooted in longstanding disinvestment in low-income, minority communities, shaped by policies such as redlining and hence may be viewed as a form of structural racism [56,57]. Substandard housing, in turn, creates environments with more asthma triggers [9,11], exacerbates symptoms and limits the impact of clinical care [58].

Participants also pointed to the limited effectiveness of healthy housing policies such as smoke-free housing. This mirrors findings from cities like Philadelphia, New York, and Norfolk, where smoke-free policies have not consistently reduced secondhand smoke exposure [59,60,61].

Both caregivers and HCWs emphasized the need for stronger enforcement of housing codes and accountability for landlords and housing authorities—including through legal action. Although HUD mandates compliance with Housing Quality Standards, enforcement often falls short [62,63,64].

Participants also noted the need to support landlords, who face barriers such as poor communication with housing authorities, difficulty accessing program information, and bureaucratic inefficiencies like delayed inspections and burdensome paperwork [65,66]. These challenges hinder timely responses to tenants and limit program participation.

## 6. Implications for Policies on Mitigating Asthma Triggers in Subsidized Housing

Our findings align with prior research indicating that, despite multiple healthy housing initiatives, current policies, and practices—particularly those intended to promote safer and healthier indoor environments—remain insufficient [11,22,38]. By examining caregivers’ lived experiences in attempting to mitigate environmental asthma triggers in the home, this study adds qualitative depth to existing quantitative and policy-focused analyses. This section further seeks to identify caregiver- and provider-informed recommendations to strengthen programs and policies aimed at reducing environmental asthma triggers in subsidized housing.

### 6.1. Housing Code Enforcement and Landlord Accountability

Participants stressed the need for stronger enforcement and greater accountability for landlords and housing authorities when unsafe conditions persist. While legal and complaint processes exist, many described them as slow, ineffective, and unresponsive. Caregivers and HCWs noted that even when tenants follow the proper steps, repairs are often delayed or ignored, prolonging exposure to asthma triggers.

Our findings suggest that residents in subsidized housing frequently find their repair requests disregarded by landlords and property managers. After repeated, unsuccessful attempts to seek action, only a few caregivers were able to pursue legal recourse—typically after being referred to nonprofit legal services by healthcare providers. For low-income families already juggling childcare, work, and other demands, navigating these complex processes often felt overwhelming and inaccessible.

The evidence presented here reinforces recommendations for proactive inspection programs to ensure safer housing conditions [38]. Policies that rely solely on tenant-initiated complaints place a disproportionate burden on families in subsidized housing and fail to produce timely remediation. These findings underscore the need for stronger housing code enforcement and more proactive regulatory oversight of landlords to ensure timely remediation in subsidized housing. Several cities have adopted more proactive enforcement strategies. For example, New York City requires landlords in buildings where a tenant has asthma to conduct annual inspections and remediate issues like mold and pests [67], and similar programs have been implemented in Baltimore, Columbus, Los Angeles, and Detroit. Additional policy strategies, such as establishing clearer remediation timelines with consistent enforcement and expedited inspection pathways may further strengthen compliance and improve housing conditions [38,68,69].

### 6.2. An Integrated Model for Healthy Housing Policy Planning and Implementation

Our results suggest that it is important to build an integrated model for policy planning and implementation to better address housing-related health disparities and prevent childhood asthma. One possibility, as our respondents suggested, is the establishment of a housing “liaison” position—such as a social worker, community health worker, or similar professional—who might help bridge the gap between residents and housing agencies. Such a role could be embedded within housing authority or local healthy home programs to streamline communications, ensure that residents’ needs and perspectives are reflected in policy and service delivery (for example, ensure that remediation requests are tracked and resolved), and connect families with community and health resources, as well as legal referrals.

The findings also highlighted the critical role that healthcare workers play in linking families to resources and helping them navigate complex healthcare and social care systems, including those related to housing and legal assistance. Medical management of asthma is undermined by poor housing conditions, providing a clear rationale for healthcare programs to be involved in providing services such as air purifiers and pest abatement. In addition, medical-legal partnerships could be incorporated into policy and program planning to strengthen families’ ability to secure necessary repairs and protections. For example, Cincinnati, Ohio, has demonstrated the potential of integrating clinicians, researchers, legal professionals, and tenant associations to advocate for healthier housing [70].

At the same time, many respondents argued that it is essential to provide support to landlords and property managers to help them meet health and safety requirements. Such support could include simplifying administrative paperwork and offering technical and logistical assistance. Supporting landlords in this way could enhance compliance and foster a more collaborative approach to maintaining healthy housing.

### 6.3. From Evidence to Practice: Scaling and Sustaining Healthy Homes Interventions

Grant-funded demonstration projects and studies have shown that multicomponent, community-based HH programs can reduce asthma triggers, decrease healthcare costs, and were feasible to implement on a large scale [71]. Yet, our work provides further evidence that these programs have not been consistently implemented.

Our respondents identified several factors that contribute to this inconsistency. First, political and fiscal pressures have led to instability and unpredictability in funding and implementation. Second, local administrative capacity may also be crucial for maintaining these programs, especially in communities with the greatest need.

Our respondents were unanimous that sustainable implementation of HH programs requires policy change. Consistent funding mechanisms, rigorous enforcement and access to legal assistance, community involvement, and wide dissemination are key elements of the types of HH programs they envisage. Together, these elements represent the policy infrastructure necessary to move HH interventions from isolated projects to sustainable, equitable public health strategies.

## 7. Limitations

Several limitations should be considered when interpreting this study’s results. First, the study was limited to public housing and subsidized housing in DC. Consistent with qualitative research standards, we emphasize transferability through thick description, providing a basis for naturalistic and inferential generalizability and for identifying essential elements that may inform interpretation across contexts [72]. Accordingly, the transferability of the findings depends on the extent to which similar structural–behavioral dynamics exist across subsidized housing settings. In addition, behavioral and lifestyle factors—such as medication adherence, chronic stress, and family routines—are known to significantly influence pediatric asthma outcomes [28,73]. To deepen interpretation, future research should examine how such factors interact with housing conditions to shape pediatric asthma management.

## 8. Conclusions

This study underscores the persistent challenges caregivers and community HCWs face in managing asthma triggers within public housing environments. The findings reveal a significant disconnect between policy intentions and on-the-ground realities and provide many thoughtful directions for policy and programs changes to strengthen housing codes, ensure access to effective HH programs, and ultimately to improve children’s health. Improving housing conditions has considerable potential to reduce the burden of asthma among vulnerable pediatric populations. Future research and policy efforts should prioritize bridging the gap between the housing regulations on the books and their practical application in subsidized housings to ensure better health outcomes for children with asthma. In addition, future research should examine low-income unsubsidized housing to better understand barriers to effective asthma mitigation, as these households often face similar environmental challenges that are further complicated by unstable housing and higher out-of-pocket costs [74].

## Figures and Tables

**Table 1:** Summary of Key Themes.

Caregivers (*N* = 22)	*N* & %	Key Quotes	Healthcare Workers (*N* = 8)	*N* & %	Key Quotes
Theme 1. Prevalence of Home Environmental Asthma Triggers					
Rodents, roaches, other Pests	13 (59.1%)	“The house was infested with mice, cockroaches, everything you could think of flying” (CG 104)	The prevalence of home environmental triggers	8 (100.0%)	“Parents will often talk about pests. Cockroaches, mice, water damage, and mold are common triggers. Carpet removal is also a major one” (HCW 7)
Mold	10 (45.5%)	“The bathroom? The mold was everywhere” (CG 108)			“Parents open certain cabinets or closets, and then some roaches like out” (HCW 6) “Old buildings hold dust and cause health problems” (HCW 3)
Carpeting, water leaks and poor ventilation	15 (68.2%)	“The carpet triggered the boy’s allergies” (CG 120)			
Aging building infrastructure	13 (59.1%)	“The building has been up since the thirties” (CG 107) “We can’t barely breathe in it” (CG 106)	Impact on children’s asthma health	6 (75.0%)	“Home environment plays a huge role in asthma outcomes and other health related outcomes” (HCW 4)
Building Infrastructural Issues	13 (59.1%)	“it’s a plumbing issue inside my building… there’s nothing we could do about it unless they tear the crap down” (CG 112)	Clinical limitations amid environmental hazards	7 (87.5%)	“it’s really hard to treat families and then send them home where they have a lot of exposure—like carpet or bad ventilation systems. It gets very challenging knowing that there’s only so much you can do to help out a family” (HCW 2)
Secondhand Smoking Exposure	7 (31.8%)	“Having to walk the kids through the building full of smoke and stuff” (CG 120)			
Impact on children’s asthma health outcome	14 (63.6%)	“He (her son) was in ICU for asthma… the housing conditions have something to do with it” (CG 108)			
Theme 2. Gaps in Implementation of Healthy Homes Services					
Awareness on Heathy Home services	19 (86.4%)	“No, I’ve never heard of that”	Landlords’ lack responses	8 (100.0%)	“They have unresponsive landlords” (HCW 9) “It is frustrating that families on vouchers tend to have the most issues and have to go back and forth with the landlord and then not trying to fix things” (HCW 4)
Landlords’ lack responses and lack of follow-ups to resident requests	13 (59.1%)	“I’ve been telling him everything, sent him lists and he never came to do nothing” (CG 117) “They don’t follow up to ensure the things get done” (CG 101)			
Inadequate inspection and pest control	13 (59.1%)	“I haven’t had an inspection since 2019” (CG 118) “They have pest control on the list. But no, never have I heard about anything like that” (CG 112)			
Unreplaced air filters	11 (50.0%)	“They haven’t changed it (air filter) since I’ve lived here. About four years”			
Theme 3. Ineffective Implementation of Healthy Home Services					
The ineffective implementation of pest control	11 (50.0%)	“Your house is treated for pests and the one next door to you doesn’t. Because it’s so close together, you’re still going to have them regardless” (CG 101)	Ineffective implementation	7 (87.5%)	“Instead of actually removing it (mold), they just cover it” (HCW 6) “Infestations keep coming back” (HCW 6)
Ineffective enforcement of “Smoking-Free” policies	8 (36.4%)	“That’s a terrible law nobody abides by it. If you go to any specific property, you will still see smoke outside” (CG 102)	Gaps in access to funded HH Programs	4 (50.0%)	“For D.C. families right now, we’re not offering referrals to any specific program” (HCW 7)
Theme 4. Challenges in Implementing Healthy Home Service					
Challenges faced by landlords	6 (27.3%)	“They’re old. How can the DC government help them, help me” (CG 108)	Geographic inequalities of housing environment challenges	4 (50.0%)	“Kids In ward 7/8 with housing issues- their asthma tends to be worse” (HCW 6)
Structural inequalities for marginalized neighborhoods	15 (68.2%)	“Just apartments in general with black kids” (CG 122) “Being a Ward 8 resident. That’s the barrier”			
Theme 5. The Multilevel Impact of Substandard Housing					
Feelings of powerlessness and vulnerability	14 (63.6%)	“It’s just a big disregard” (CG 107). “If you ain’t got enough money. You’re not going to be seen or heard” (CG 112)	Caregivers’ powerlessness and vulnerability	7 (87.5%)	“It’s emotional—for them and for me” (HCW 7)
Frustration and distrust	14 (63.6%)	“They just take your money and don’t provide the services that’s required” (CG 107)			
Legal actions	8 (36.4%)	“I had to get a lawyer so that I could move.” (CG 120)			
Theme 6. Suggestions for Healthy Home Services					
Strengthening accountability and oversight of landlords	14 (63.6%)	“Stay on top of these landlords then you wouldn’t have these problems inside the household” (CG 118)	Listening to families’ voices	7 (87.5%)	“Allows the parent to have a voice is very important” (HCW 2)
			Ensuring sustainable funding	5 (62.5%)	“The city should establish a budget so this can be done sustainably” (HCW 4) “Need more money—invest up-front to fix these houses rather than pay higher health-care costs later” (HCW 7) “they’re trying to get some grants so that program can start back up” (HCW 8)
			Addressing the cycle of inequality	7 (87.5%)	“Affordable units aren’t affordable, voucher holders still can’t move, the cycle continues” (HCW 6) “Data show asthma is rising due to poor housing, that evidence can drive better laws” (HCW 8)
			Policies reinforcing landlord responsibilities	8 (100.0%)	“They need to be stricter on the landlord—really enforcing the rules” (HCW 2) “We are moving past the point where community partners can shoulder this—the city needs to budget for it”, and “landlords feel more forced to act when it’s the city, not just a clinic, telling them” (HCW 4) “Research can help put in better laws for landlords to stay on top of their buildings” (HCW 8)
			Supports for landlords/property managers	5 (62.5%)	“City officials should collaborate with landlords—see what barriers they have and give support, not just another fine” (HCW 4) “Some landlords want to fix problems but lack the money—the city should help, not just fine them” (HCW 5) “Building relationships with housing resources can help landlords finds lower-cost fixes, benefiting families and owners” (HCW 8)
			Innovative Approaches	4 (50%)	“There could be smoke detector” (HCW 5)
			Caregiver Advocacy	5 (62.5%)	“Advocate for these families. Children are our future; we need to help them as much as we can” (HCW 9)

## Data Availability

The qualitative interview data from this study are not publicly available due to confidentiality and privacy restrictions outlined in the Institutional Review Board (IRB) approval. Participants were caregivers from subsidized housing communities and healthcare providers and sharing raw transcripts or recordings could compromise confidentiality. However, the data are available from the corresponding author upon reasonable request, subject to ethical approval. De-identified excerpts relevant to the study findings are included within the manuscript.
